# Thermal-Insulation Effect and Evaluation Indices of Asphalt Mixture Mixed with Phase-Change Materials

**DOI:** 10.3390/ma13173738

**Published:** 2020-08-24

**Authors:** Biao Ma, Heting Shi, Jiayun Xu, Kun Wei, Xiaoqing Wang, Yue Xiao

**Affiliations:** 1 Key Laboratory for Special Area Highway Engineering of Ministry of Education, Chang’an University, Xi’an 710064, China; shting@chd.edu.cn (H.S.); xujiayun@chd.edu.cn (J.X.); Weikun@chd.edu.cn (K.W.); wangxiaoqing716@163.com (X.W.); 2 Hunan Provincial Communications Planning, Survey & Design Institute Co., Ltd., Changsha 410200, China; leonxiaoy@163.com

**Keywords:** road engineering, asphalt mixture, phase-change thermal-insulation agent, thermal-insulation effect, evaluation indices

## Abstract

Under strong winds and at low temperatures, heat loss of hot-mix asphalt mixtures is likely to occur, which leads to temperature segregation. Temperature segregation affects the forming quality and the performance of asphalt pavements. In this study, a phase-change thermal-insulation agent (PCTIA) was prepared for reducing the temperature dissipation. A cooling simulation experiment was performed to test the temperature-dissipation process for an ordinary asphalt mixture and the asphalt mixture mixed with PCTIA (AM-PCTIA). The thermal-insulation effect was analyzed according to the temperature difference and the thermal-insulation extension time. Moreover, two indices—the thermal-insulation accumulated time difference value (IATDV) and thermal-insulation accumulated time difference index (IATDI)—were proposed for evaluating the thermal-insulation ability and efficiency. The results indicated that the temperature at the center of the AM-PATIA was 4 °C higher than that for the ordinary asphalt mixture. The insulation time was prolonged by 29.8 min at the ambient temperature of 15 °C. As the ambient temperature increased, the thermal-insulation effect of the PCTIA improved.

## 1. Introduction

When a hot-mix asphalt mixture is applied to asphalt pavement, rapid heat loss and temperature segregation are likely to occur [1,2]. These problems often arise under strong winds and at low temperatures. Temperature dissipation and segregation have adverse effects on pavement construction. For instance, the paving temperature cannot satisfy the specifications, and the effective paving and compaction time is short [3]. These problems cause cracking, looseness, rutting, and other pavement distresses, which reduce the performance and serviceability of asphalt pavement [4,5,6]. Studies have indicated that the service life of asphalt pavement is significantly shortened by temperature segregation; the serviceability and lifecycle of severely segregated pavement can be reduced by >50% [7].

Therefore, for improving the construction quality of asphalt pavements, the most crucial task is to reduce the temperature dissipation of the asphalt mixture. Considerable achievements have been made in reducing the temperature dissipation of the asphalt mixture during pavement construction. Studies have indicated that the temperature difference between the asphalt mixture and the external environment can be reduced by adding additives to the asphalt mixture [8,9]. The most commonly used additive is the warm-mix agent. The addition of warm-mix agent can reduce the construction temperature of the asphalt mixture by 20–30 °C [10,11]. This can not only extend the effective time of the asphalt mixture construction but also reduce the short-term aging degree of the asphalt mixture. Additionally, the temperature separation can be slowed by optimizing the construction technology [12]. For reducing the transportation time, it is feasible to reduce the distance between the mixing station and the paving site [13]. After the hot-mix asphalt mixture is transported to the construction site, a secondary mixing process can be conducted to make the internal temperature distribution more uniform. Most directly, the temperature dissipation of the asphalt mixture can be controlled by modifying the asphalt-mixture transport vehicle and increasing its thermal-insulation effect [14]. The simplest method is to use a canvas and quilt to cover the transport vehicle. Alternatively, an operation vehicle that integrates mixing, heating, and heat preservation can be used to improve the insulation effect [15]. The addition of heat oil in vehicles can provide heat energy and avoid the large-scale cooling of the mixture in the transportation process. Although these methods have a certain effect in the application of asphalt pavement, some of them cannot be popularized, owing to their difficulties; thus, the situation of temperature dissipation and segregation remains, especially under strong winds and at low temperatures.

Phase-change materials (PCMs) play a significant role in regulating the temperature by releasing or absorbing thermal energy during phase transitions. These materials have a high heat-storage density, large heat-storage capacity, small volume, good chemical stability, and constant temperature in the endothermic and exothermic processes [16,17]. Because of their excellent performance, PCMs have a wide range of application prospects in the field of road engineering [18,19]. Wang [20] and Tan [21] prepared composite PCMs (CPCMs) and mixed them into asphalt mixtures. The road performance of the asphalt mixtures mixed with CPCMs satisfied the requirements of specifications. The CPCMs were effective for reducing the rising and cooling rates of the asphalt mixtures. According to a comprehensive analysis of relevant studies in various fields, at present, PCMs are mainly used to adjust the temperature of the pavement in the service stage. However, the application of PCMs in transportation and construction remains scarce. If the PCMs is used in the construction process of the hot-mix asphalt mixture and make full use of the latent heat of the PCMs, the temperature dissipation of the hot-mix asphalt mixture will be alleviated.

In light of the foregoing problems, a phase-change thermal-insulation agent (PCTIA) was prepared in this study and mixed with an asphalt mixture. An environment chamber was used to test the temperature changes under the cooling condition. The temperature-dissipation process between the asphalt mixture mixed with PCTIA (AM-PCTIA) and the ordinary asphalt mixture was tested at different environmental temperatures. The temperature difference between the two asphalt mixtures at the same position was examined to analyze the thermal-insulation effect of the PCTIA. Then, the thermal-insulation extension time was calculated according to the difference between the times when the two asphalt mixtures decreased to the same temperature. Finally, two indices—the thermal-insulation accumulated time difference value (IATDV) and thermal-insulation accumulated time difference index (IATDI)—were proposed for evaluating the thermal-insulation ability and efficiency. This study provides a valuable reference for solving the problem of temperature dissipation and segregation during pavement construction in most areas.

## 2. Materials

### 2.1. Phase-Change Thermal-Insulation Agent

**Figure 1:**
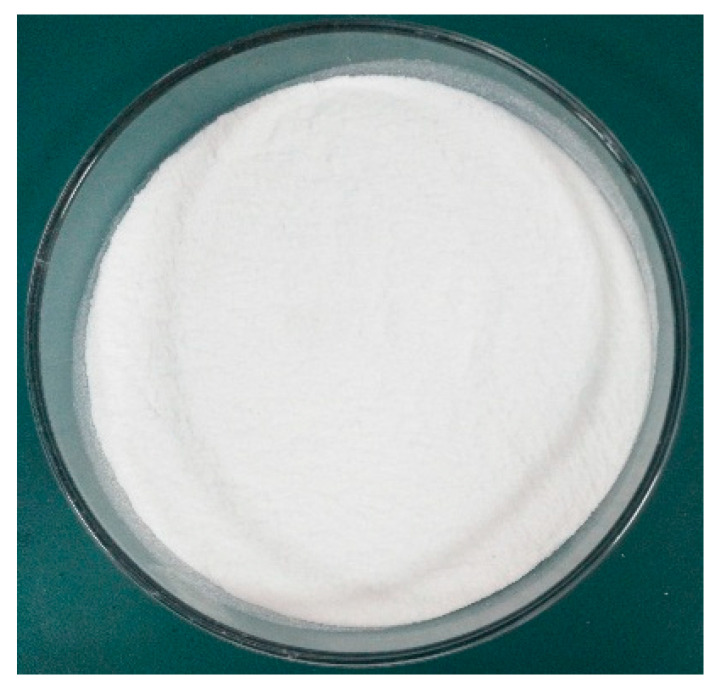
Phase-change thermal-insulation agent (PCTIA).

The PCTIA was custom-made, as shown in Figure 1. It is a white powdery particle. The particle size of the PCTIA is 0.2–0.3 mm. The PCTIA is a solid-liquid phase change material. Its main properties are as follows.

(1) Heat-storage capacity

**Figure 2:**
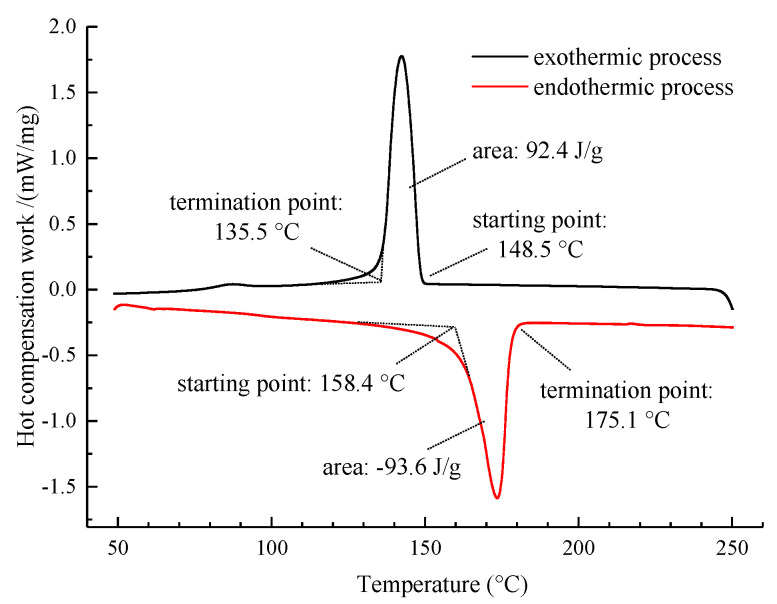
Differential scanning calorimetry (DSC) results for the PCTIA.

In the construction process of the asphalt mixture, the PCTIA must absorb and release the latent heat energy within the specific temperature domain. Moreover, a high phase change enthalpy is recommended for ensuring sufficient latent heat of the PCTIA. The differential scanning calorimetry (DSC) curves of the PCTIA are presented in Figure 2.

As shown in Figure 2, in the temperature range of 50–250 °C, there was only one endothermic peak during the endothermic process of the PCTIA, and there was one exothermic peak during the cooling process. Clearly, the PCTIA only underwent one phase change in both the endothermic and exothermic processes within the temperature range. According to the DSC curves, the enthalpy value of the PCTIA in the endothermic process was 93.6 J/g, and the phase-change temperature ranged from 158.4 to 175.1 °C. This is consistent with the asphalt-mixture discharge temperature of 140–180 °C specified in the Technical Specification for Highway Asphalt Pavement Construction (JTG F40-2004) [22]. The enthalpy value in the exothermic process was 92.4 J/g, and the phase-transition temperature ranged from 135.5 to 148.5 °C. This satisfies the recommendation of the Technical Specification for Highway Asphalt Pavement Construction (JTG F40-2004) that the asphalt compaction starting temperature should be no lower than 135 °C. Therefore, it can be inferred that the PCTIA can release latent heat during the construction of the asphalt mixture, thereby reducing the temperature dissipation and providing thermal insulation to the asphalt mixture.

(2) Heat stability

**Figure 3:**
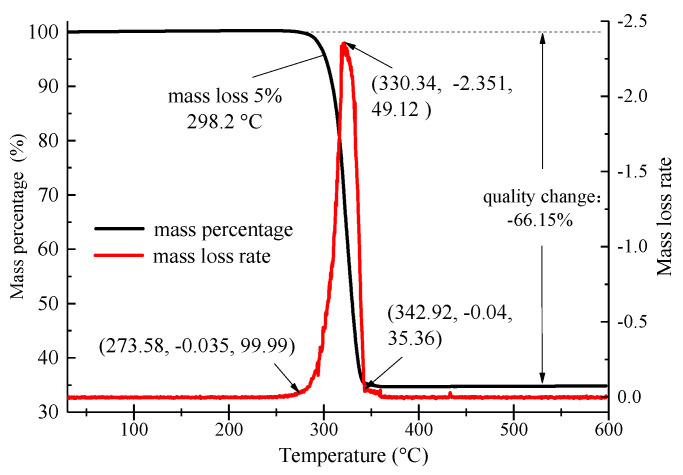
Thermogravimetric curve of the PCTIA.

The construction temperature of the hot-mix asphalt mixture was relatively high. The PCTIA mixed into the mixture endured a very high temperature. For example, the mixing temperature of the hot-mix asphalt mixture is about 170–180 °C. If the PCTIA underwent serious mass loss under high-temperature conditions, the phase-change latent heat was insufficient, reducing the thermal-insulation effect. Therefore, a thermogravimetric analysis was performed to analyze the heat stability of the PCTIA. The thermogravimetric curve is presented in Figure 3.

As shown in Figure 3, there was a small mass loss of the PCTIA at 273.58 °C. The temperature was 298.2 °C when the mass loss of the PCTIA reached 5%, which was significantly higher than the upper limit of the construction temperature. Apparently, the mass loss of the PCTIA was concentrated at 273.58–342.92 °C. Although the mass loss rate was the highest in this temperature range, it had no effect on the application in the asphalt pavement. The results indicate that the PCTIA has sufficient heat stability to be used in asphalt pavement construction.

(3) Heat conductivity coefficient

The heat conductivity coefficient is a physical quantity that reflects the capacity of heat transmission. The PCTIA mixed into the hot-mix asphalt mixture was mostly wrapped in asphalt or in contact with the aggregate. The main heat-absorption route was heat conduction. The capacity of heat transmission was determined by measuring the heat conductivity coefficient of the PCTIA. In this study, a Hot Disk (TPS 2500) Heat Conductivity Instrument was used to test the heat conductivity coefficient of the PCTIA at 170, 140, 65, 20, and −40 °C. The temperature range that can be tested of the Heat Conductivity Instrument is from −243 to 1000 °C, and the heat conductivity coefficient range is 0.005–1800 W/(m·K). The results for the heat conductivity coefficient are presented in Table 1.

As shown in Table 1, the maximum heat conductivity coefficient of the PCTIA in the test temperature range was 0.6005 W/(m·K). The thermal conductivity coefficient at 170 °C was lower than that at −40 °C after the phase change of the PCTIA.

### 2.2. Asphalt Mixture

To investigate the effect of the PCTIA on the temperature dissipation during the transportation and pavement of the hot-mix asphalt mixture, 70# asphalt was used in this study. Coarse aggregate and mechanism sand were adopted as the coarse and fine aggregates, respectively. The filler was clean and dry limestone powder, which was ground and exhibited no caking. The composition of the aggregate mixture used in this study is presented in Table 2. The asphalt and PCTIA contents were 4.84 and 2.5 wt % of the aggregate mass, respectively. The optimal contents of the PCTIA and the asphalt are determined by Marshall design method and road performance test results.

## 3. Experimental Method

To examine the temperature dissipation of the hot-mix asphalt mixture mixed with PCTIA and without PCTIA in different environments, the test was performed in an environmental test chamber. The temperature range was −40 to 150 °C, and the temperature fluctuation was <0.5 °C. For brevity, the asphalt mixture without PCTIA is referred to as the “ordinary asphalt mixture” hereinafter.

**Figure 4:**
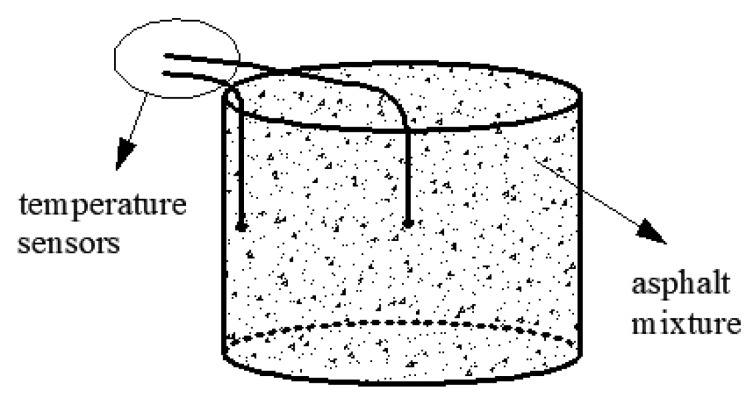
Layout of the temperature sensors.

First, 10 kg AM-PCTIA and ordinary asphalt mixture samples were taken after being mixed homogeneously and placed into the same containers in a loose state. All types of vibration were avoided after the mixture was loaded into the container. Pt100 platinum thermal resistance temperature sensors were embedded in the center and the edge of the asphalt mixture, respectively. The working temperature range of the temperature sensors is −200 to 850 °C; the accuracy is ±0.15 °C. The buried depth of the sensors remained at half of the total depth to the greatest extent possible. The temperature sensor at the edge did not contact the vessel wall. The temperature changes of the two samples were monitored in real time by the temperature sensors, and a corresponding schematic is presented in Figure 4. To avoid severe temperature dissipation in the test process, the asphalt mixture embedded with sensors was placed in the environment chamber as quickly as possible. Gaps were left between the specimens. The environment chamber was adjusted and stabilized to the required temperature range before the experiment. Four ambient temperatures—0, 5, 10, and 15 °C—were selected to study the temperature-dissipation process of the AM-PCTIA. The temperatures of the two samples decreased from 170 to 80 °C.

## 4. Results and Analysis

### 4.1. Temperature-Dissipation Process

**Figure 5:**
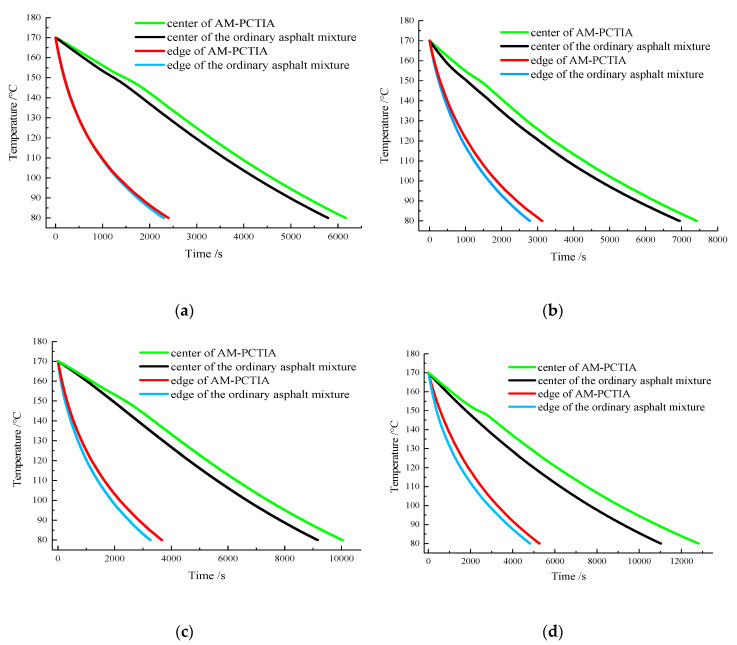
Temperature change curves of the two samples at different ambient temperatures: (**a**) 0 °C; (**b**) 5 °C; (**c**) 10 °C; (**d**) 15 °C.

The simulation results for the temperature-dissipation process of the ordinary asphalt mixture and the AM-PCTIA at different ambient temperatures are presented in Figure 5.

As shown in Figure 5, during the temperature decrease of the two samples from 170 to 80 °C, the temperatures at the center and edge of the two samples gradually decreased over time. The cooling rate decreased with a decrease in the sample temperature. The cooling rate of the ordinary asphalt mixture was higher than that of the AM-PCTIA at the same position, indicating that the AM-PCTIA had the function of thermal insulation. At different ambient temperatures, the temperature at the center was higher than that at the edge for both samples, and the cooling rates at the centers of both samples were relatively low. This is because the ambient temperatures were lower than the mixture temperature, and the mixture at the edge exchanged heat with the external environment rapidly through the container wall directly. In comparison, the asphalt mixture at the center was far from the edge; thus, the heat transfer was relatively slow.

According to the cooling curves of the AM-PCTIA, the temperature-dissipation process was divided into the following three stages.

(1) In the first stage, the overall temperature of the mixture was higher than the initial exothermic temperature of PCTIA (148.5 °C). Therefore, the PCTIA did not undergo a phase transformation and released heat in the form of sensible heat.
(2) In the second stage, with a reduction in the overall temperature of the mixture, the PCTIA underwent a phase change and released a large amount of latent heat, which provided thermal insulation to the mixture. The cooling rate of the mixture decreased significantly; thus, there were small platforms in the curve.
(3) In the third stage, the latent heat of the PCTIA was completely released, and the heat energy was released in the form of sensible heat. As the temperature of the samples decreased, the temperature difference and the cooling rate between the samples and the environment gradually decreased.

### 4.2. Temperature Difference

**Figure 6:**
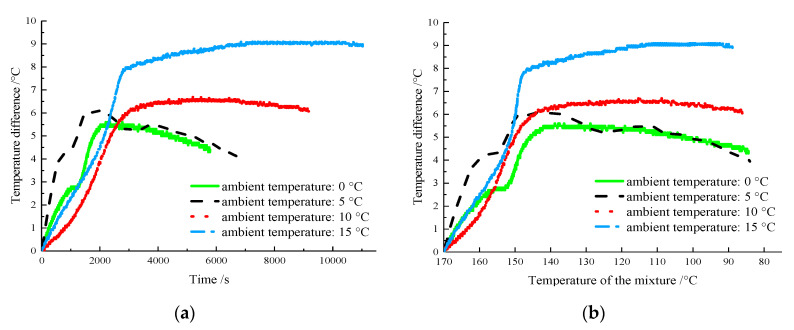
Variation of the temperature difference between the centers of the two samples at different ambient temperatures: (**a**) relationship between temperature difference and time; (**b**) relationship between temperature difference and temperature.

The temperature differences between the centers of the AM-PCTIA and the ordinary asphalt mixture at different ambient temperatures are presented in Figure 6.

As shown in Figure 6a, the temperature difference increased over time in the period of 0–2500 s. After 2500 s, the temperature difference at 10 and 15 °C tended to be stable, whereas that at 0 and 5 °C tended to decrease. At the ambient temperature of 15 °C, the maximum temperature difference of the mixture was 9.1 °C, whereas it was 5.6 at 0 °C, indicating that the maximum temperature difference increased with the ambient temperature. This is because when the heat-transfer rate of the mixture decreased as the ambient temperature increased, the latent heat effect of the PCTIA became more obvious. When the temperature of the ordinary asphalt mixture reached 80 °C, the temperature difference between each value of ambient temperature was >4 °C, confirming that the effect of the PCTIA on the temperature was significant. As shown in Figure 6b, when the temperature of the AM-PCTIA at different environmental temperatures was >145 °C, the temperature difference between the two samples increased rapidly. At the ambient temperatures of 0, 5, 10, and 15 °C, the final temperatures in the rapidly increasing temperature difference section of the samples were 142, 143, 140, and 146 °C, respectively; thus, the temperatures were still in the phase-change temperature range in the exothermic stage.

To further analyze the foregoing phenomena, the reasons for the rapid increase in the temperature difference in the initial period of the mixture cooling were identified as follows. (1) The addition of PCTIA reduced the heat conductivity of the asphalt mixture. According to previous studies [23,24,25], the heat conductivity coefficients of most ordinary asphalt mixtures are in the range of 1.5–2.5 W/(m·K). On the basis of Section 2 of this paper, the maximum heat conductivity of the material was 0.61 W/(m·K), which was far lower than the heat conductivity of the ordinary asphalt mixture. Therefore, the temperature dissipation of the mixture slowed with the addition of the PCTIA. (2) The AM-PCTIA near the edge underwent a phase transition and released the latent heat sooner than that at the center. Additionally, it provided heat energy to the surrounding mixture, reducing the cooling rate of the mixture at the center and resulting in a large temperature difference between the samples. (3) The PCTIA at the center released the latent heat gradually with the decreasing temperature, which reduced the temperature dissipation of the mixture and increased the temperature difference.

### 4.3. Thermal-Insulation Extension Time

**Figure 7:**
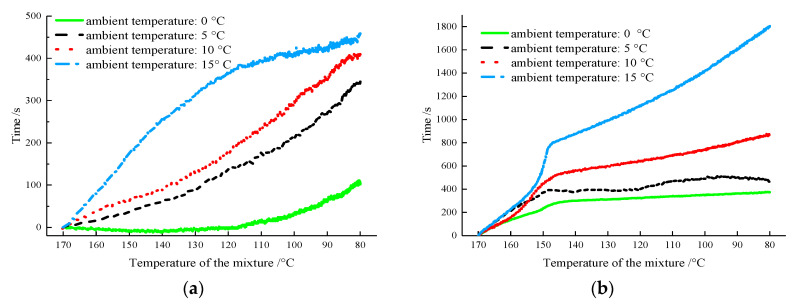
Change in the thermal-insulation extension time during the cooling processes of the two samples: (**a**) edge; (**b**) center.

At the same position and ambient temperature, the difference between the times taken for the AM-PCTIA and the ordinary asphalt mixture to decrease to the same temperature was defined as the extended thermal-insulation time, and it is shown in Figure 7.

As shown in Figure 7, with an increase in the ambient temperature, it took longer for the two samples to decrease to the same temperature at the same position. This is because the total amount of phase-change latent heat in the asphalt mixture remained unchanged, and the release rate of the phase-change latent heat decreased with the increasing ambient temperature. The mixture at the edge exchanged heat with the external environment first, and the rate of heat loss was higher here than that at the center; thus, the thermal-insulation extension time at the edge was significantly lower than that at the center. To analyze the time difference between the two samples at the same temperature under different ambient temperatures more distinctly, temperatures of 160, 140, 120, 100, and 80 °C were selected. Then, the time differences corresponding to five temperatures between the AM-PCTIA and the ordinary asphalt mixture under different ambient temperatures were calculated. The results are presented in Table 3.

As indicated by Figure 7 and Table 3, when the temperatures at the edges of the two samples decreased from 170 to 160, 120, and 80 °C, the maximum time differences were 78, 366, and 447 s, respectively. When the temperature decreased from 170 °C to the different temperatures, the maximum time difference at the center was three times or more that at the edge. Consider the ambient temperature of 15 °C as an example: it took 1792 s longer (four times longer) for the temperature at the center of the AM-PCTIA to decease to 80 °C than that at the edge of the ordinary asphalt mixture. Clearly, the PCTIA played a significant role in reducing the temperature dissipation of the asphalt mixture, which ensured that the temperature requirement of the asphalt mixture was satisfied at the beginning of the rolling. Additionally, the extension of the thermal-insulation time provided sufficient time for the transportation and rolling of the pavement.

### 4.4. Evaluation Indices of Thermal-Insulation Effect

The objective of this study was to investigate the comprehensive thermal-insulation effect of the PCTIA on the asphalt mixture. In a previous work [26], it was proposed that the latent heat accumulation value and latent heat regulation index could be used as evaluation indices for the thermoregulation effect of PCMs. On the basis of these two indices, according to the requirements of the paving and rolling times of the mixture in the construction process, the IATDV and IATDI were proposed. These two indices take into account not only the latent heat in phase transition on the thermal insulation effect, but also the influence of sensible heat on the temperature of the asphalt mixture.

**Figure 8:**
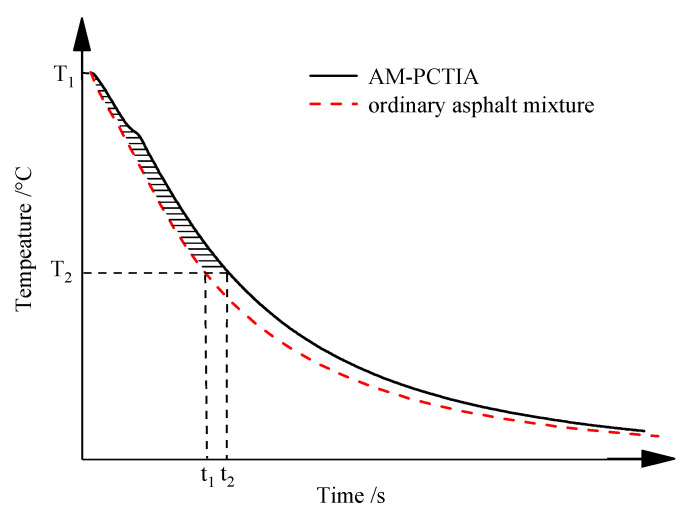
Cooling processes of the two samples.

Similar to the calculation method for the latent heat accumulation value proposed by our research group in the early stage of research [26], the IATDV was determined by calculating the integration of the time difference within the temperature range of the phase-change thermal insulation, as indicated by the shadow area in Figure 8.

The calculation formula for the IATDV is as follows:

(1) $$\mathrm{IATDV}=\int_{T_{1}}^{T_{2}}|f\left(T\right)-y(T)|dT\approx \sum \Delta T_{i}\times \Delta t_{i}$$

where *f*(*T*) represents the function of the temperature–time relationship curve for the AM-PCTIA, *y*(*T*) represents the function of the temperature–time relationship curve for the ordinary asphalt mixture; *T* represents the temperature (°C) of the asphalt mixture; *T*_2_ represents the initial temperature of the mixture; *T*_1_ represents the terminal temperature of the mixture; *t* represents the testing time (s); *t*_1_ represents the testing time of the ordinary asphalt mixture; *t*_2_ represents the testing time of the AM-PCTIA; and Δ*T_i_* and Δ*t_i_* represent the temperature and time differences of the two samples, respectively.

The IATDV reflects the thermal-insulation ability of the PCTIA in the asphalt mixture. It is mainly composed of two parts: the time difference caused by the release of phase-change latent heat and the time difference caused by the change in the thermal parameters of the asphalt mixture due to the presence of the PCTIA. In this study, 170 °C was selected as the initial temperature. The terminal temperature of the IATDV can be selected according to the actual demand.

The formula for calculating the IATDI is presented in Equation (2). It can be used to evaluate the thermal-insulation efficiency of the PCTIA in the asphalt mixture.

IATDI = IATDV/(Δ*t* × Δ*T*) (2)

Here, Δ*t* represents the thermal-insulation time domain of the PCTIA, and Δ*T* represents the thermal-insulation temperature domain of the PCTIA.

In this study, 140, 120, 100, and 80 °C were selected as the terminal temperatures for the calculation of the IATDV and IATDI of the asphalt mixture. The results are presented in Table 4.

**Figure 9:**
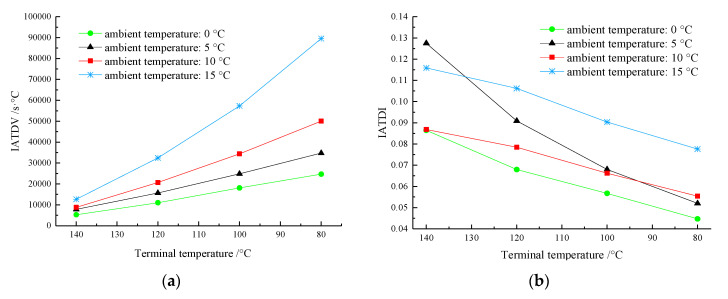
Two indices of the asphalt mixture mixed with PCTIA (AM-PCTIA) at different ambient temperatures: (**a**) IATDV; (**b**) IATDI.

For a more intuitive understanding, the relationship curves between the two evaluation indices and the terminal temperature at different ambient temperatures are shown in Figure 9.

As shown in Figure 9a, in the cooling process of the asphalt mixture at different ambient temperatures, the IATDV increased gradually with a decrease in the terminal temperature. This is because the ordinary mixture was not mixed with the PCTIA; thus, the temperature dissipation was very fast. The temperature difference between the AM-PCTIA and the ordinary mixture increased gradually, leading to an increase of the integral. When the temperatures of the two samples of the asphalt mixture decreased to the same value, the IATDV increased with the ambient temperature. When the temperature of mixture decreased to 80 °C at an ambient temperature of 15 °C, the IATDV was 89,537.8 s·°C, which was 0.8 times higher than that at 10 °C. A higher ambient temperature corresponded to a stronger thermal-insulation ability of the PCTIA.

As shown in Figure 9b, at the same ambient temperature, the IATDI decreased with a decrease in the terminal temperature, indicating that the thermal-insulation effect of the PCTIA decreased gradually. Before the temperature of the asphalt mixture decreased to 120 °C, there was no obvious regularity of the IATDI, but after the temperature of the mixture decreased below 120 °C, the IATDI was clearly the highest when the ambient temperature was 15 °C, compared with the other three ambient temperatures. The comparison revealed that the IATDI of the PCTIA was the highest when the ambient temperature was 15 °C. Thus, the thermal-insulation efficiency was the highest when the material was used at 15 °C.

## 5. Conclusions

(1) A PCTIA for the construction of a hot-mix asphalt mixture was prepared. The phase-change temperature range of the PCTIA in the endothermic and exothermic process matched the construction demand, and the PCTIA had good heat stability and low heat conductivity.
(2) The temperature-dissipation processes of the ordinary asphalt mixture and AM-PCTIA were simulated through a cooling experiment. The thermal-insulation effect was relatively strong at the center of the asphalt mixture, and the temperature of the AM-PATIA was 4 °C higher than that of the ordinary asphalt mixture. The insulation time was prolonged by 29.8 min for the center of the asphalt mixture at the ambient temperature of 15 °C.
(3) Two evaluation indices (the IATDV and IATDI) were proposed for evaluating the thermal-insulation ability and efficiency of the PCTIA. In summary, the IATDV in the same temperature range increased with the ambient temperature, indicating that the thermal-insulation ability of the PCTIA increased with the ambient temperature. The PCTIA had the best thermal-insulation effect and the highest thermal-insulation efficiency at the ambient temperature of 15 °C.

## Figures and Tables

**Table 1:** Heat conductivity coefficients of the PCTIA at different temperatures.

Temperatures (°C)	−40	20	65	140		170
Heat conductivity coefficient (W/(m·K))	0.5694	0.5765	0.5852	0.6005 (endothermic process)	0.5648 (exothermic process)	0.5441

**Table 2:** Composition of the aggregate mixture.

Sieve Size/mm	16	13.2	9.5	4.75	2.36	1.18	0.6	0.3	0.15	0.075
Passing rate/%	100.0	96.0	76.5	53.0	37.0	26.5	19.0	13.5	10.0	6.0

**Table 3:** Time difference corresponding to five temperatures.

Environmental Temperature	Position	Time Difference/s				
		160 °C	140 °C	120 °C	100 °C	80 °C
0 °C	Edge	−3	−9	0	30	105
	Center	135	300	327	351	375
5 °C	Edge	15	63	138	213	342
	Center	215	440	463	540	477
10 °C	Edge	36	87	180	294	408
	Center	156	553	638	734	865
15 °C	Edge	78	255	366	411	447
	Center	223	872	1114	1410	1792

**Table 4:** Date of the thermal-insulation accumulated time difference value (IATDV) and thermal-insulation accumulated time difference index (IATDI).

Ambient Temperature	Relevant Parameter	Terminal Temperature (°C)			
		80 °C	100 °C	120 °C	140 °C
0 °C	Temperature domain (°C)	90	70	50	30
	Time domain (s)	6150	4575	3270	2115
	IATDV (s·°C)	24,685.4	18,110.4	11,046.6	5302.2
	IATDI	0.0447	0.0567	0.0679	0.0864
5 °C	Temperature domain (°C)	90	70	50	30
	Time domain (s)	7422	5220	3450	2055
	IATDV (s·°C)	34,762.3	24,842.9	15,679.4	7861.4
	IATDI	0.0520	0.0680	0.0909	0.1275
10 °C	Temperature domain (°C)	90	70	50	30
	Time domain (s)	10036	7419	5278	3380
	IATDV (s·°C)	50,071.0	34,391.3	20,701.5	8807.6
	IATDI	0.0554	0.0662	0.0784	0.0869
15 °C	Temperature domain (°C)	90	70	50	30
	Time domain (s)	12816	9066	6115	3656
	IATDV (s·°C)	89,537.8	57,345.5	32,468.5	12,706.0
	IATDI	0.0776	0.0904	0.1062	0.1158
